# The Role of Different Thoughts in Tacit Coordination and Its Malleability by Interventions

**DOI:** 10.5334/joc.503

**Published:** 2026-05-26

**Authors:** Lionel Newman, Ming Cao, Susanne Tauber, Marieke van Vugt

**Affiliations:** 1 Bernoulli Institute of Mathematics, Computer Science & Artificial Intelligence, University of Groningen, Groningen, NL; 2 Engineering and Technology Institute, University of Groningen, Groningen, NL; 3 Department of Political Sociology, University of Amsterdam, Amsterdam, NL

**Keywords:** Reasoning, Social cognition, Attention

## Abstract

Previous work has shown that successful tacit coordination depends on the availability of working memory resources. Much less is known about how thought processes, such as mind-wandering and self- vs. other-oriented attention, influence tacit coordination. In this experiment, we examined the effect of these thought processes on tacit coordination, and whether we could influence performance and thought processes through brief meditation interventions. While coordination and thought processes were not significantly affected by the interventions when compared to a control intervention, we found that thought processes, as measured by thought probes randomly interleaved between trials, significantly influenced coordination performance. Specifically, dyads performed significantly better when other-oriented than when self-oriented, showing greater convergence on shared decision rules over time. This study demonstrates the importance of orienting attention beyond self-generated responses in order to mutually adapt actions and mental states to achieve shared goals in iterated social coordination.

## 1. Introduction

Humans are often able to make decisions and take actions that account for others’ mental states in order to coordinate actions and achieve joint goals. This occurs in a wide variety of social contexts, including during business negotiations for terms of agreement, in team movements during sports, and when deciding where to meet with new friends. While cognitive scientists have recently begun to shed light on the mechanisms necessary for social coordination, the associated subjective thought processes remain largely unexplored.

Subjective thoughts have recently started to be studied under the umbrella of mind-wandering research (Smallwood & Schooler, 2015). This research proceeds by inserting self-report questions about the current thoughts into the cognitive task, at intervals of approximately 30–90 seconds. It then becomes possible to relate the responses to these thought probes to objective measures such as behavioural performance, physiology or brain activity. Some key findings include a reduction in accuracy when participants report to be mind-wandering (e.g., Jin et al., 2019; Smallwood & Schooler, 2015), as well as a reduction in evoked potential magnitude (e.g., Jin et al., 2019; Kam et al., 2011; Smallwood et al., 2008). A common finding in this field is that people mind-wander approximately 40% of the time (Smallwood & Schooler, 2015), and this persists even in challenging tasks such as the Complex Working Memory task (Huijser et al., 2018). While most of the studies of mind-wandering have focused on the task-relatedness of subjective thought, there are also some studies examining how other aspects of subjective thoughts, such as self-relatedness (e.g., Ruby et al., 2013) or phenomenological stickiness (Yang & van Vugt, 2025) affect task performance and brain activity.

Most research on subjective thought has focused on the same paradigms, such as a go-nogo task or simple working memory tasks (Smallwood et al., 2011). Very little is known about the role of subjective thought in social cognition. The best clue for how subjective thought could be involved in social cognition comes from the fact that social cognition relies crucially on working memory operations, which we also know are strongly related to subjective thought (e.g., Schooler et al., 2011). Specifically, cognitive control of working memory plays a causal role in the ability to selectively process a single perspective; either one’s own perspective without interference from the other’s perspective, or the other’s perspective without interference from one’s own perspective. When a person is engaged in off-task thinking, it is more difficult to process the other’s perspective. Indeed, in our previous work, we found that matching image choices occurred more frequently and within fewer trials under a low working memory load control condition compared to high load imposed by a concurrent *n*-back task (Newman et al., 2024). Further evidence for the importance of working memory was provided by the finding that better performance was significantly associated with higher P3 amplitude time-locked to image onset—an EEG measure classically associated with encoding to working memory (Donchin, 1981; Donchin & Coles, 1988). Similar to prior single-participant studies, our study demonstrated that representing the mental states underlying others’ behaviour is cognitively demanding. Working memory plays a causal role such that mental state attribution and interpersonal coordination performance diminish as fewer resources are available when loaded by a concurrent working memory task.

Another aspect of subjective thought that may be relevant for social cognition is the thoughts’ self-relatedness. Although there is, to our knowledge, no research on the topic, it is highly likely that when one spends more time thinking about the other, then social coordination improves. This would be particularly true for forms of social coordination in which speech is prohibited. A good example of this is tacit coordination, the ability to coordinate actions without explicit communication. In our previous work on tacit coordination (Newman et al., 2024), we used a task in which dyads viewed unique images in each trial, and attempted to select the same image as each other without communication, and were shown both their responses at the end of each trial. They needed to observe the other and attribute underlying mental states, and also dynamically update their own mental states and actions in order to align their behavioural decisions. They do so by mutually attributing intended decision rules from previous image selections and using these attributions to predict and plan subsequent selections. In other words, this relies on other-directed subjective thought. Participants typically converged on shared decision rules over time (such as selecting the darkest image, the reddest, or the image containing certain angles or shapes). This alignment was reflected in matching image choices.

In addition to examining how tacit coordination depends on subjective thought, even stronger evidence for their relationship could be provided by manipulating subjective thought through interventions. A promising area of interventions on subjective thoughts are contemplative practices such as meditation (e.g., Requier et al., 2024). Focused Attention Meditation (FAM), which involves deliberately sustaining attention on a single object such as the experience of breathing while disengaging from other experiences (Dahl, Lutz, & Davidson, 2015), has been shown to reduce off-task thinking. For example, in Mrazek et al. (2013), training in FAM led to increased working memory capacity, with improved performance on a cognitively-demanding reading comprehension task mediated by reduced mind-wandering. And, relevant for tacit coordination, which requires substantial manipulation of social information in working memory, FAM training has frequently been shown to improve working memory capacity and selective encoding of relevant information to working memory (Chiesa, Calati, & Serretti, 2011; Jha et al., 2019; Quach, Jastrowski Mano, & Alexander, 2016), These FAM-induced working memory enhancements are associated with reduced off-task thought and reduced reactivity to distractions (Cahn & Polich, 2009; Jain et al., 2007; Mrazek, Franklin, Phillips, Baird, & Schooler, 2013). FAM-induced improvements in working memory are also associated with increased P3 amplitude during encoding of task-relevant information to working memory (Incagli, Tarantino, Crescentini, & Vallesi, 2020; Lomas, Ivtzan, & Fu, 2015; Malinowski, 2013; Wang et al., 2020). This association between FAM-induced change in P3 and FAM-induced change in working memory performance is relevant to our previous study discussed above where we found that higher P3 amplitude was associated with more availability of working memory resources as well as enhanced behavioural performance in a cognitively-demanding social coordination task (Newman et al., 2024).

Importantly, improvements in cognitive performance have also been reported for studies implementing a single, brief (~10 minute) bout of training (for a review, see: Leyland, Rowse, & Emerson, 2019). Similarly, Jankowski & Holas (2020) found improvements in attention performance after a short FAM training. Furthermore, the reported changes in the P3 have been observed even for brief (~10 minute) FAM training (Bokk & Forster, 2022). Together, these findings suggest that FAM is likely to lead to improvements in participants’ ability to remain on-task in order to selectively process task-relevant information and resist interference from distractors, and that improvements are even possible for short interventions.

Another contemplative practice that could influence tacit coordination is loving kindness meditation (LKM). A single bout of brief (10 minute) LKM has been shown to improve social connectedness (Hutcherson, Seppala & Gross, 2008). If this is the case, then it is likely that this practice should increase the amount of thought devoted to the other person, which in the case of tacit coordination is also likely to improve task performance.

Taking together the research discussed so far, the goal of this study is to test whether performance in an iterated pure coordination task is related to on-task thought and other-oriented thought, and whether it can be influenced with a brief meditation intervention. We hypothesized that coordination performance would be improved with on-task thinking and with more other-focused thought. We also expected that FAM would result in more on-task thought, LKM would result in more other-oriented thought, and that both would thus improve coordination performance.

## 2. Methods

### 2.1 Participants

116 participants aged 18–32 years old (M = 21.2, SD = 2.74) took part in the study. Since coordination differs between same-sex and mixed-sex dyads (Baker et al., 2016; Cheng, Li, & Hu, 2015), participants were randomly matched into 58 same-sex dyads (16 male-male pairs, 42 female-female pairs). All participants reported having normal or corrected-to-normal vision, passed a color blindness test, and reported having no history of neurological injury or illness.

To prevent confounding by coordination effects that have been previously associated with intimate relationship (Goldstein, Weissman-Fogel, Dumas, & Shamay-Tsoory, 2018; Kinreich, Djalovski, Kraus, Louzoun, & Feldman, 2017; Reindl, Gerloff, Scharke, & Konrad, 2018), we ensured dyads were not friends, romantic partners, or family. Upon arrival to the location of the study, participants briefly introduced themselves to each other before experimental setup.

In total, participants took approximately one hour to complete the experiment which included approximately 10 minutes for questionnaires, 40 minutes for the behavioural task, and 10 minutes for the intervention. The experiment and recruitment took place at the University of Groningen. Procedures were approved by the Research Ethics Committee (CETO, approval number 87774858). All participants provided written informed consent and were compensated with a base rate of €10 per hour, plus a bonus of up to €4 depending on performance in order to introduce a motivation for coordination (see Supplementary Materials).

### 2.2 Coordination Task

Dyads performed a computerized pure coordination task in the same room while seated at separate tables, each with its own monitor (set at identical resolution) and separate keyboard. Visual contact was blocked by a floor-to-ceiling bookcase, and participants were instructed not to communicate verbally during the task, which was monitored by an experimenter.

**Figure 1:**
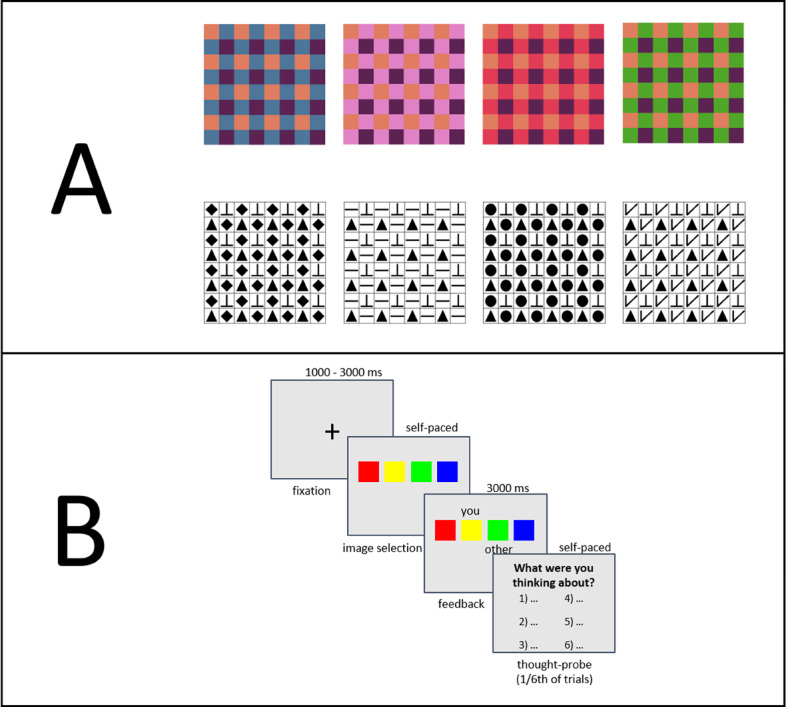
Example Stimuli and Trial Sequence. *Note*. Panel A shows an example of colour and shape image sets shown in a trial. In each trial, a set of four previously-unseen images (either colour or shape) is presented, from which participants try to select the same image. Panel B shows trial sequence.

The task consisted of two blocks, 90 trials each, separated by a 10-minute intervention and interleaved with occasional thought-probes to measure self-generated thought (see below). Each block was preceded by onscreen instructions and practice trials (see Supplementary Material). In each trial, participants viewed four novel images composed of alternating shapes or colours (examples in Figure 1), and were instructed to select the same image as their co-actor. The stimuli were adapted from Alberti, Sugden, & Tsutsui (2012), and this task was also used in our previous study (Newman et al., 2024). The coordination task and stimuli in this study are identical to that used in our latter study, except that the shape stimuli were simplified to minimize the chance that performance would be significantly diminished in shape as compared to colour stimuli, as was the case in Newman et al. (2024). Examples of shape and colour images are shown in Figure 1A. The images displayed in each trial were arranged on a horizontal plane, with left-to-right order randomized separately for each participant. Participants were informed of this so that their selections were based on image features rather than positions. Responses were self-paced and at the end of each trial the images selected by both participants were displayed for 3 seconds, allowing participants to become familiar with their co-actor’s choices. Trial sequence is shown in Figure 1B.

An important aspect of our task is that coordination is initially difficult as participants have little information about which image the other participant is likely to choose, and coordination performance typically improves over trials as participants encode information during the feedback phase of each trial regarding image selections. Performance improvement over trials is thought to reflect convergence on shared decision rules (i.e., focal points), with each participant inferring which image features their co-actor intends to choose based on previous selections, and adjusting their choices accordingly. For example, based on both participants’ previous selections, they might eventually decide to select the darkest image, the image with the most red, or the image containing a certain shape, angle, or line orientation which was present in previous selections. Thus, this task serves as a simplified but empirically tractable method for operationalizing multi-agent interactions where each individual’s previous actions mutually influence their subsequent actions, ideally towards alignment of intentions, beliefs, and behaviours.

To prevent emergent decision rules from carrying over between pre-intervention and post-intervention blocks, one block used images made of alternating colours while the other used images made of alternating shapes. This resulted in a 2 (colour vs. shape stimuli) by 2 (pre- vs. post-intervention block) by 3 (intervention types, detailed below) between-subjects design. Stimulus type order (i.e., colour block first vs. shape block first) and intervention type were counterbalanced across dyads.

## 2.3 Interventions

Each dyad was randomly assigned to one of three intervention groups: Focused Attention Meditation (FAM; 19 dyads), Loving Kindness Meditation (LKM; 20 dyads), or Neutral Control (NEU; 19 dyads). Interventions were administered by pre-recorded audio, played through headphones which were identical in model and set to an identical volume level for both participants in each dyad. Interventions lasted for approximately 10 minutes and all were voiced by the same male guide, and delivered in English.

The interventions were based on those used in previous studies. The FAM consisted of instructions to focus on sensations of breathing and was based on the recording used in Ma *et al*. (2021) and Colzato *et al*. (2016). The LKM consisted of instructions to generate feelings of warm wishes toward and from loved ones, friends, and neutral acquaintances while visualizing these individuals, and the script was based on Seppala *et al*. (2014). The NEU intervention was taken from the same study, consisting of instructions to visualize details of neutral scenes such as a bookstore and a parking lot. Scripts for the interventions are included in Appendix B, and audio recordings are available in the online repository (see Data and Code Availability section).

We did not include manipulation checks since self-report measures of the effectiveness of meditation interventions are fraught with problems (e.g., Grossman, 2011, 2019), and hence we decided not to use those here to test effectiveness of the intervention.

### 2.4 Thought Probes

To measure subjective thoughts during the task, we inserted thought probes after a randomly-defined set of trials that accounted for 1/6^th^ of all trials (15 out of 90 trials per block), The probe and response options were adapted from previous work on self-generated thought which coded on-task vs. off-task thought (Huijser, van Vugt, & Taatgen, 2018; Stawarczyk, Majerus, Maj, Van der Linden, & D’Argembeau, 2011; Unsworth & Robison, 2016), to which we added a self-focused vs. other-focused dimension. The probe consisted of the question, “What were you thinking about before you chose an image?” with the following response options:

1. I thought about which image to choose based on what the other person would choose [coded as “on-task: other-focused”]
2. I thought about which image to choose without basing my decision on what the other person would choose [“on-task: self-focused”]
3. I was evaluating aspects of the task (e.g., my performance, how long it takes, difficulty of the task) [“off-task: task-related interference”]
4. I was distracted by my environment (sound/temperature, etc.) or by my physical state (hungry/thirsty) [“off-task: external distraction”]
5. I was daydreaming/I thought about task-unrelated things [“off-task: mind-wandering”]
6. I was not paying attention, but I did not think about anything specific [“off-task: inattentiveness”]

Participants had an unlimited amount of time to respond, and were instructed at the beginning of the task that their responses to these probes would not affect their performance score or payment. One thought probe was presented in practice trials at the beginning of the task.

### 2.5 Questionnaires

Prior to the task, each participant completed two questionnaires. First, the Interpersonal Reactivity Index (IRI; Davis, 1983) which is a validated measure of four dimensions of trait empathy: perspective taking, fantasy, empathic concern, and personal distress. Second was the validated 18-item version of the Five Facet Mindfulness Questionnaire (FFMQ-18), which is a short version with higher reliability than the original 39-item version (Medvedev, Titkova, Siegert, Hwang, & Krägeloh, 2018) and measures five subscales of trait mindfulness: acting with awareness, describing, non-judgmental, non-reactive, and observing.

Participants also answered brief questions about experience with meditation. Specifically, they answered whether they had ever tried meditation, whether they had ever kept a regular (near-)daily practice for at least one month, and whether they had a daily practice at the time of the experiment. For dyad-level analyses, prior experience with meditation was coded as 0 for neither participant having experience, 1 for one participant having experience, and 2 for both having experience; current daily practice was coded using this procedure as well. Also for dyad-level analyses, the mean score was calculated for IRI and FFMQ as well as the mean of each subscale. The purpose of these questionnaires was to control for inter-individual differences in trait empathy, trait mindfulness, and meditation experience, which we predicted would influence coordination performance and thought probe responses.

### 2.6 Data Analysis

#### Interaction-specific effects on performance

Following previous investigations of focal point coordination, “matching frequency” and “coordination index” were calculated as summary statistics for performance (Alberti et al., 2012; Bardsley, Mehta, Starmer, & Sugden, 2010; McMillan, Rascovsky, Khella, Clark, & Grossman, 2012; Mehta, Starmer, & Sugden, 1994). Matching frequency is the proportion of correctly matched choices between paired, interacting participants. Coordination index is the probability that two non-interacting, non-paired participants, chosen at random from the entire sample, select matching responses on a given set of four images (that is, a given trial). This baseline represents the null hypothesis that image selections were not based on dyad-specific interactions and instead were driven by the stimuli themselves. A matching frequency significantly greater than the coordination index indicates a positive contribution of dyad-specific interaction to performance, over and above the contribution of stimuli alone.

To compute the coordination index, a sample distribution was generated by a permutation test. Specifically, 1,000 samples were generated by randomly selecting with replacement the choices of two non-interacting participants on each image set. The distribution of mean correct in these generated samples was compared against the empirical matching frequency. A matching frequency greater or equal to the 0.95 quantile of coordination index indicates matching frequency significantly exceeds the coordination index (α = 0.05).

#### Controlling for confounding variables

Before analysing relationships between meditation interventions, coordination performance, and thought probe responses, we first checked for effects of possible task-related covariates such as stimulus type (colour or shape), block number (block 1 or 2), and time of experimental session (hour of the day). In addition, to account for a heterogeneous sample of participants in terms of demographics, we also tested for effects of age, sex, education level, English level (see Supplementary Materials for details on numeric coding of education and English levels), and prior meditation experience (see above).

First, we tested for confounding factors affecting overall coordination performance. Since performance is a dyad-level measure, for each dyad we calculated the dyad-level means for age, education level, and English level, and for prior meditation experience we coded whether either or neither subject had ever meditated, ever maintained a near-daily meditation practice, and currently maintained a near-daily practice at the time of the experiment. Then a simple linear regression was fitted separately for each variable, with mean performance (defined as the proportion of trials with correctly matched image choices) as the outcome variable. For block and stimulus type, we opted to fit linear mixed-effects models using maximum likelihood (with dyad as a random intercept) to maximize power since grouping was possible with two values per dyad (i.e., two blocks). Significance for these linear mixed-effects models was determined using chi-square test and Akaike information criterion (AIC). Specifically, we performed a Type III test in which the full model was compared to a reduced model containing identical terms but excluding the predictor of interest. All other models mentioned in this paper used the same Type III comparison approach.

We then repeated this procedure to determine effects of these possible confounding factors on other outcome measures, namely the difference in mean performance from pre- to post-intervention blocks as well as the difference in proportion of on-task and other-focused thought probe responses from pre- to post-intervention. After identifying which confounding variables significantly affected which outcomes, we controlled for them in subsequent analyses on the respective outcome variable (see below). We did this using a two-step hierarchical modelling approach, whereby confounding variables were included as independent variables in both steps, but the independent variable of interest (e.g., intervention) was excluded from the first step for F-test model comparison. All linear regressions were implemented using the “lm()” function in R, linear mixed-effects models using “lmer()” from the “lme4” package, and model comparisons using “anova()” (R Core Team, 2021).

We also examined nonlinear changes over tine using Generalized Additive Models (GAMs) in R (“gam()” function in the “mgcv” package) (Wood, 2006). The GAM is a flexible technique that captures linear and nonlinear relationships (for details, see Supplementary Materials; for a tutorial, see van Rij *et al.*, 2019). We opted to use this method because, as is common in time-series data, changes in performance across trials—the learning process that participants undergo in converging towards shared choices—were not linear.

#### Effects of interventions on pre-post change in coordination performance

To determine whether dyad coordination performance was significantly affected by intervention, we started for simplicity with a linear regression with the dependent variable defined as the difference in mean performance from pre- to post-intervention blocks for each dyad, and with intervention type as an independent variable at step two of the hierarchical modelling procedure. Dummy variables for FAM and LKM were created so that their respective model coefficients reflected their effects against NEU. We tested for coefficient inflation due to multicollinearity by computing the Variable Inflation Factor (VIF) using the “vif” function contained in the “car” package in R, and repeated this for each of the models described below (Craney & Surles, 2002; Akinwande, Dikko, & Samson, 2015).

#### Effects of interventions on self-generated thought

In order to determine the effect of interventions on self-reported frequency of various thoughts, we fitted a LMM for on/off-task and a separate LMM for self/other-orientation. Each LMM used pre-post intervention differences in proportion of thought-probe response as the outcome variable, and used the same fixed and random effects as the LMM described above for mean dyad performance, but using individual subject-level instead of dyad-level measures for prior experience with meditation, current daily practice, and longest daily practice, as well as using a random intercept for subject instead of dyad. We verified each model using the same selection procedures described above, comparing each LMM to a reduced model which excluded intervention type as a fixed effect.

#### Relationships between coordination performance and subscales of mindfulness and empathy

To determine whether mindfulness and empathy related to performance in the coordination task, a LMM was fitted to predict mean dyad performance using mean dyad score in each questionnaire’s subscale as a fixed effect, in addition to the other variables included in models of mean dyad performance previously discussed. Again, the model was compared to a reduced model that excluded questionnaire subscale score.

#### Bayesian statistics

At various points, we complement the Frequentist statistics with Bayesian statistics. We do so because Bayesian statistics, computed using the BayesFactor package (Morey & Rouder, 2015), allow us to not only obtain evidence in favour of the alternative hypothesis, but also evidence in favour of the null hypothesis. We used the default settings for the BayesFactor package. In interpreting the results of the Bayes Factors, we used the conventional thresholds proposed by Kass & Raftery (1995). Specifically, we assume that Bayes Factors between 0.3 and 3 are not worth a bare mention; Bayes Factors between 0.1 and 0.3, and between 3 and 10, are substantial evidence; Bayes Factors smaller than 0.1 or larger than 10 are strong evidence.

## 3 Results

### 3.1 Demographics

Before launching into the main analyses, it is important to establish whether the intervention groups are comparable. Supplementary Table 5 compares the intervention groups on demographics, questionnaires, and meditation experience. None of the p-values (for one-way ANOVA or chi-square test of independence) are significant (all p > 0.07), and a Bayes Factor analysis shows that the groups are equivalent (BF_10_ < 0.27) on all variables, except for education. For education, the Bayes Factor of 1.00 indicates that the data are too uncertain to establish whether there is a difference or equivalence between the groups.

### 3.2 Coordination Index

To establish that performance in the coordination task was driven by dyad-specific interaction rather than by properties of the stimuli themselves, we compared performance to baseline coordination indices computed by a permutation test for shape and colour stimuli. Matching frequency for both shape stimuli (0.75) and colour stimuli (0.77) were above the 0.95 quantile of the coordination indices (shape: 0.41; colour: 0.39), indicating that performance was driven by dyad-specific interactions rather than stimulus properties.

### 3.3 Identifying confounding variables

We first tested whether demographic covariates affected mean dyad performance. A significant effect of block was found (χ^2^(1) = 26.35, p < 0.001; ΔAIC = 24.35), whereby performance in block 2 increased by 10.8% compared to block 1. Thus, subsequent analyses on mean performance controlled for block number.

### 3.4 Effects of on-task thinking on coordination performance

We then asked whether coordination performance was influenced by whether participants were focused on the task or not. First, we tested for confounding variables affecting mean thought probe responses, namely proportion of on-task (inverse of off-task) and other-focused (inverse of self-focused) response. Mean on-task thought significantly decreased with age (Adjusted R^2^ = 0.047, F(1, 114) = 6.65, p = 0.011; β = –0.011), significantly decreased in participants with any meditation experience (Adjusted R^2^ = 0.025, F(1, 114) = 4.00, p = 0.048; β = –0.048), significantly decreased in participants who regularly meditated before (Adjusted R^2^ = 0.10, F(1, 114) = 13.90, p < 0.001; β = –0.11), and significantly decreased in participants with current regular meditation practice (Adjusted R^2^ = 0.057, F(1, 114) = 7.77, p < 0.01; β = –0.15). Therefore, we controlled for age, any previous meditation experience, any previous regular meditation experience, and current regular meditation practice in analyses of on-task thought. Contrary to our expectations, no effect of on-task thought was found (either participant on-task vs. both off-task: χ^2^(1) = 0.70, p = 0.40, ΔAIC = 1.3; both on-task vs. either off-task: χ^2^(1) = 1.00, p = 0.32, ΔAIC = 1.0).

**Figure 2:**
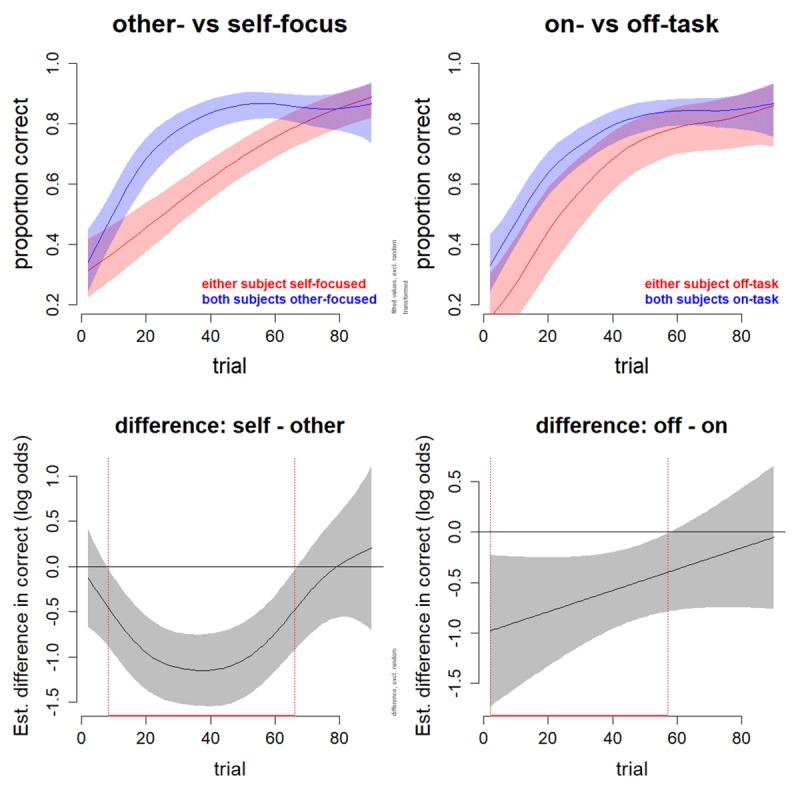
Effects of Thought Probe Responses on Performance Over Trials. *Note*. Hierarchical GAM analysis controlling for block revealed a significant effect of other-focus on performance over trials (χ^2^(3) = 13.708, p < .001, ΔAIC = 22.21), both for the parametric coefficient referenced to self-focus (Z = 4.605, SE = 0.140, p < .001, estimated coefficient = 0.64) and the nonlinear interaction between other-focus and trial (χ^2^(3.336) = 19.91, p < .001). For on-task, no significant effect was found (χ^2^(3) = 3.208, p = .093, ΔAIC = 5.97). Shading represents pointwise 95% confidence intervals. Black horizontal lines in the bottom panel represent log-odds values of 0 (i.e., no difference between estimates for other- and self-focus or on- and off-task), with red lines indicating the trial window of significant difference (self- vs. other-focus: trials 8–66; on- vs. off-task: trials 2–57). Full model outputs are shown in Supplementary Tables 2 and 3.

In addition to testing whether on-task thought affected mean dyad performance, we also tested whether it affected changes in dyad performance over trials using GAM analyses. We controlled for block by fitting a reduced model—containing block as a parametric term and a smooth term for the interaction between block and trial—and comparing it to a full model—containing identical terms plus a parametric term for thought-probe response (on-task: both on-task vs. either off-task) and a smooth term for the interaction between thought-probe response and trial. The full model was not significantly better than the reduced model (χ^2^(3) = 3.208, p = 0.093, ΔAIC = 5.97), with no significant interaction between on-task and trial (χ^2^(1.001) = 2.07, p = 0.15, but a significant effect of the parametric coefficient for on-task referenced to off-task (Z = 2.84, std. error = 0.187, p < 0.001, estimated coefficient of 0.53); Figure 2; for full model outputs see Supplementary Table 3). This reflects a significant linear (but absence of nonlinear) benefit of on-task over off-task thought for performance change over trials, with this benefit estimated to be significant up to trial 57 out of 90 trials per block (Figure 2).

### 3.5 Effect of other-focused thought on performance

**Figure 3:**
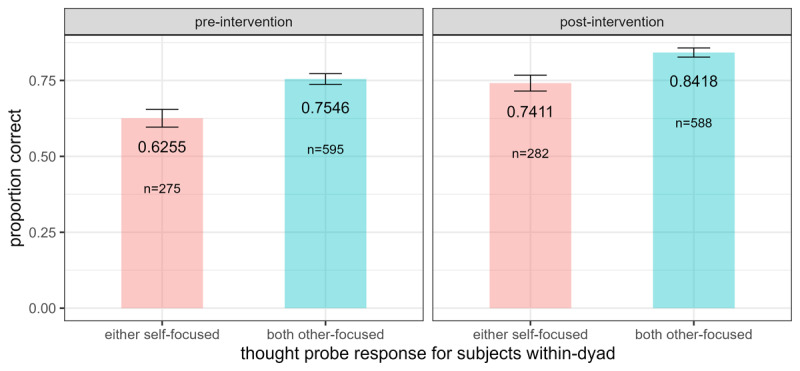
Other-Focus Enhances Coordination Performance. *Note*. Hierarchical logistic regression modelling controlling for block (pre- vs. post-intervention) revealed a significantly higher likelihood for dyads to successfully coordinate on trials where both participants were other-focused compared to trials where either participant was self-focused (χ^2^(1) = 27.04, p < 0.001, ΔAIC = 25.0); “n” represents number of trials included in analysis; error bars represent standard error of the mean.

We then asked whether coordination performance was affected by other-focused thinking. Disregarding intervention and controlling for block, dyads were significantly more likely to choose the same image on trials where either participant within-dyad was other-focused, compared to trials where both participants were self-focused, as revealed by post-hoc model comparison between full and reduced logistic regressions using a chi square test (χ^2^(1) = 26.09, p < 0.001, ΔAIC = 24.1). Similarly, successful coordination was significantly more likely on trials where both participants within-dyad were other-focused compared to when either was self-focused (χ^2^(1) = 27.04, p < 0.001, ΔAIC = 25.0; Figure 3).

In addition to analyzing effects of thought probe responses on mean dyad performance, we also performed an exploratory test of their non-linear effects on dyad performance across trials. Using a logistic GAM, we tested whether thought probe response had a significant effect on change in accuracy over trials. For each of the two thought probes (self- vs. other-focused and on- vs. off-task), we fitted a model using thought probe response and block as parametric (non-spline) predictors representing main effects, and we implemented the nonlinear interactions that account for change in accuracy over trials using “smooth” terms. A one-dimensional nonlinear regression line is fitted for smooth terms for each level of the thought probe. Four smooth terms were estimated in our GAM: 1) The interaction between trial and thought probe (fitting a nonlinear relationship between trial and thought probe response); 2) The interaction between trial and block (fitting a nonlinear relationship between trial and block); 3) A smooth term for trial (representing the average learning curve across all conditions), and 4) A random factor smooth to account for variation between dyads (analogous to random slope in standard LMMs). We then compared each model with a corresponding reduced model that contained identical terms except for those containing thought probe response.

For other-focus, a chi-square test on the restricted maximum likelihood scores revealed the full model was significantly better than the reduced model (χ^2^(3) = 13.708, p < 0.001, ΔAIC = 22.21), with a significant effect of both the parametric coefficient for other-focus referenced to self-focus (Z = 4.605, std. error = 0.140, p < 0.001, estimated coefficient of 0.64) and a significant interaction between other-focus and trial (χ^2^(3.336) = 19.91, p < 0.001; Figure 2; for full model outputs see Supplementary Table 2). This reflects significantly greater linear and nonlinear improvement in performance over trials during other-focus compared with self-focus. For on-task vs. off-task, no significant effect was found (χ^2^(3) = 3.208, p = 0.093, ΔAIC = 5.97).

### 3.5 Effects of trait empathy and mindfulness on performance and thought-probes

We then asked whether individual differences in trait empathy influenced coordination performance and thought probes. No significant correlation was found between mean dyad questionnaire measures and mean dyad performance nor pre-post differences in performance. Testing whether this was due to lack of evidence or evidence in favour of the null hypothesis, Bayes Factor analyses revealed anecdotal to moderate evidence supporting the null hypothesis across questionnaire measures for both mean performance and pre-post differences in performance (Supplementary Table 4).

Analysing correlations between participant questionnaire scores and mean participant thought-probe responses, a significant negative correlation was found between the “non-reactive” subscale of the FFMQ and mean other-focus (r(114) = –0.20, p = 0.032; BF_10_ = 1.13), such that participants who were more non-reactive reported less other-focus. Yet, the Bayes Factors indicate a lack of evidence for both of these effects.

For correlations between participant questionnaire scores and change in thought-probe responses from pre- to post-intervention, a significant negative correlation was found between the “perspective-taking” subscale of the IRI and change in on-task thought (r(114) = –0.20, p = 0.033; BF_10_ = 1.13), such that participants who report a higher tendency to spontaneously adopt the viewpoint of others were less on-task after interventions compared to before.

For the rest of the questionnaire measures, no significant correlations were found, and Bayes Factor analyses yielded anecdotal to moderate evidence supporting null hypotheses for both mean and pre-post differences in performance, on-task thought, and other-focused thought (Supplementary Table 4).

### 3.6 Confounding variables for intervention effects

Next, testing for confounding variables affecting performance difference from pre- to post-intervention, we found a significant effect of sex (Adjusted R^2^ = 0.064, F(1, 53) = 4.7, p = 0.035) with females improving 9.30% more than males at post-intervention (3 dyads were excluded from this analysis as their sex was not reported). Pre-post accuracy difference was also significantly affected by time of experiment (Adjusted R^2^ = 0.052, F(1, 56) = 4.10, p = 0.048) with post-intervention performance improvement diminishing by hour of the day (β = –0.017). Finally, prior meditation experience—when either participant within a dyad had any previous meditation experience—did not significantly improve post-intervention performance (Adjusted R^2^ = 0.066, F(1, 56) = 3.95, p = 0.052). No effects were found for either participant within-dyad having ever had a regular meditation practice or having a regular practice currently; however, within the LKM group specifically, prior regular meditation practice significantly diminished post-intervention performance improvement by 10.90% (Adjusted R^2^ = 0.19, F(1, 18) = 5.33, p = 0.033). In sum, since pre-post change in performance was significantly affected by sex, time of experiment, and prior regular meditation practice, we controlled for these variables in subsequent analyses on pre-post performance differences.

Finally, we tested for confounding factors affecting pre-post changes in proportion of on-task and other-focused thought. Current regular meditation practice significantly decreased pre-post change in on-task thought (Adjusted R^2^ = 0.041, F(1, 114) = 5.96, p = 0.016, β = –0.14), such that participants who regularly practiced meditation reported being significantly less on-task at post-intervention than pre-intervention. No confounding variables were found to affect pre-post changes in other-focused thought. Thus, we controlled for current regular meditation practice in analyses on pre-post changes in on-task thought.

### 3.4 Effects of interventions on performance and thought-probe responses

**Figure 4:**
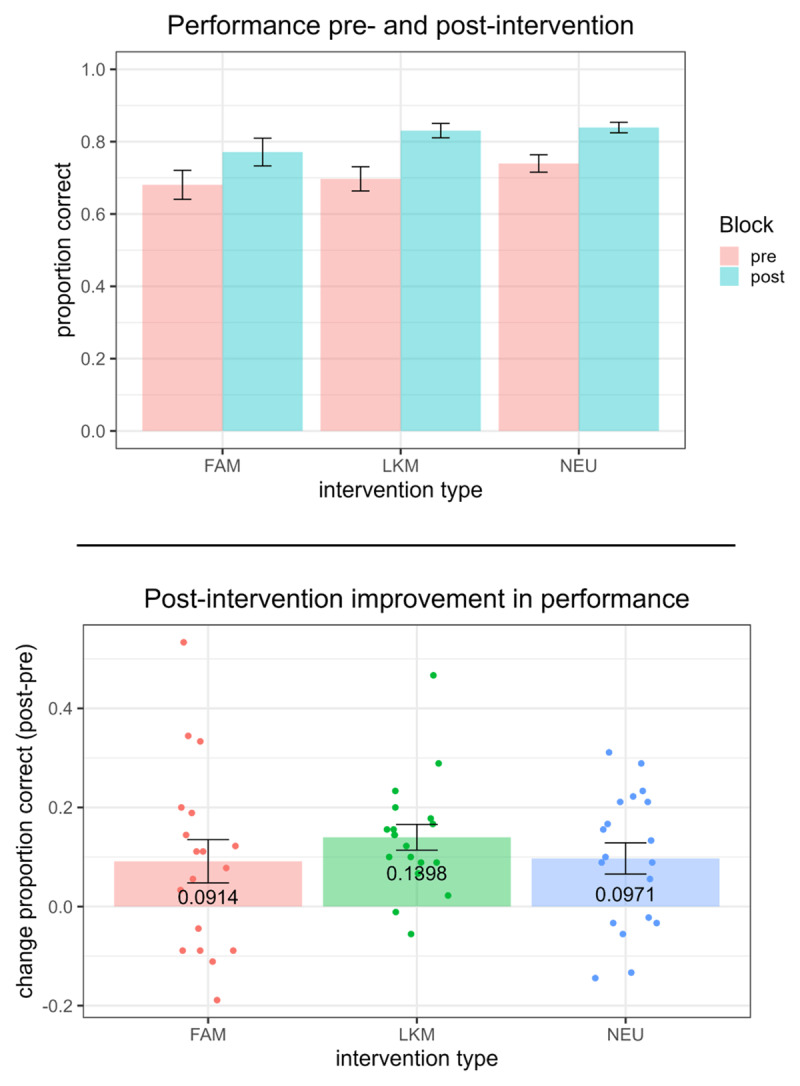
Coordination Performance Pre- and Post-Intervention. *Note*. No significant effect of intervention type on change in performance from pre- to post-intervention was found. Bayes Factor analysis revealed moderate evidence in favor of the null hypothesis (BF_10_ = 0.22). Top panel shows mean dyad performance. In bottom panel, points represent change in performance for each dyad. Error bars represent standard error of the mean. FAM = focused attention meditation; LKM = loving kindness meditation; NEU = neutral control.

Using hierarchical regression modelling, we found no significant effect of intervention type on change in performance from pre- to post-intervention when correcting for significant covariates (Adjusted R^2^ change = –0.013, F(1, 2) = 0.61, p = 0.55; Figure 4). To test whether this non-significant finding was due to lack of evidence or evidence supporting the null hypothesis, we calculated the Bayes Factor of full model terms against null model terms, and found moderate evidence in favour of the null hypothesis (BF_10_ = 0.22), demonstrating significant evidence for no difference between the interventions on the change in performance. One possible explanation for a lack of significance could be collinearity. However, this was not the case, since a VIF analysis showed no significant multicollinearity among predictors for this and subsequent models discussed below (see Table 6 in Supplementary Materials).

Apart from effects of interventions on performance, we also tested for effects on thought probes. No significant effect of intervention type was found for pre-post change in participant on-task thought (Adjusted R^2^ change = –0.0071, F(3, 111) = 0.57, p = 0.57) nor change in other-focused thought (Adjusted R^2^ = –0.0078, F(2, 111) = 0.56, p = 0.57). Testing whether this was due to lack of evidence or evidence in support of the null hypothesis, Bayes Factor analyses revealed moderate evidence in favour of the null hypothesis for both on-task (BF_10_ = 0.13) and other-focus (BF_10_ = 0.13), indicating that the interventions did not change these aspects of thought.

## 4. Discussion

In this study, we predicted that higher rates of on-task thought would predict better coordination performance. However, we found no significant effect of mean rate of on-task thought on coordination. Interestingly, exploratory time-series analysis using GAMs indicated a significant linear benefit of on-task over off-task thought for performance change over trials, with this benefit estimated to be significant for the first 57 out of 90 trials per block. This suggests that task demands may shift over trials, with on-task thought playing a more important role in initial trials compared to later trials. However, this effect on initial trials was not enough to drive a significant effect of mean on-task thought. Incidence of off-task thought in our participants was unusually rare (mean participant on-task response rate was 92.6%) compared to previous studies using similar thought probes where on-task thought ranged from approximately 40% to 60% (Huijser et al., 2018; Stawarczyk et al., 2011; Unsworth & Robison, 2016). It is possible that participants were highly motivated during this task due to the social and game-like aspects. Therefore, ceiling effects may have limited our findings. Another possible limitation is that, due to the structure of our task in which focal points emerge over time, on-task thought may not be crucial for later trials after focal points have already been established. As a consequence, some off-task responses may reflect successful performance in later trials, thus diminishing any on-task effects on performance in earlier trials—especially given that the low number of off-task trials gives them an outsized influence when occurring during successful coordination. A valid criticism of our approach, therefore, is that performance on trials before and after focal point emergence may reflect different processes, where during later trials more on-task thinking may reflect a process similar to “overthinking”. We also hypothesized that coordination performance was significantly higher when both interacting participants engaged in other-focused thought as opposed to one being self-focused, as well as when one interacting participant was other-focused as opposed to both being self-focused. We also found that participants converged on decision rules over trials significantly faster when other-focused. This finding contributes to a growing body of work suggesting that individuals are able to coordinate on joint tasks by integrating information about their co-actor’s actions with their own actions, even during turn-based tasks where actions take place in rounds and not continuously in real-time. For example, Sacheli et al. (2021) found that co-actor errors in a joint, turn-based button pressing task elicited responses classically associated with self-generated errors. Crucially, these error responses were induced not only when the co-actor failed to achieve the joint goal (pressing the incorrect button), but also when the co-actor successfully achieved the goal using unexpected behaviour (pressing the correct button but using the incorrect finger), indicating that interacting agents track not only goal information but also the actions taken by a co-actor. Our results provide evidence that subjective thoughts tracking a co-actor’s actions in this way are beneficial in iterated pure coordination scenarios. We speculate that this finding may indicate that in such iterated pure coordination scenarios, mutual alignment is likely to be more effective than emergent role distribution (i.e., leader-follower configuration). A leader-follower structure can emerge when one participant consistently performs actions according to self-defined decision rules while the co-actor consistently infers intentions from the other’s behaviour (King, Johnson, & van Vugt, 2009). Although our thought probes did not directly ask participants whether they were attempting to lead or follow, we interpret higher proportion of self-focused image selection as likely to reflect strategic leading rather than, e.g., carelessness on the part of participants, particularly in light of the high proportion of on-task responses as well as monetary incentive to perform optimally. Anecdotally, some participants informed experimenters after the end of the task that they attempted to lead in this way, leading us to check whether self-focused responses were high in certain participants, which may reflect leading behaviour. Interestingly, while the average self-focused response rate across participants was 11.6%, the self-focused response rate among the 12 most self-focused participants ranged from 39.6% to 86.6%. This appears to be intentional given that this group reported to be on-task with a mean rate of 90.1% (SD = 12%). Our findings suggest that these likely attempts to create a leader-follower structure were significantly less successful than a strategy of mutual alignment. While this is the first study, to our knowledge, indicating this advantage of mutual alignment in an iterated pure coordination game, it is worth noting that findings in other tasks are mixed, sometimes showing a benefit of mutual alignment (Curioni, Vesper, Knoblich, & Sebanz, 2019; Konvalinka, Vuust, Roepstorff, & Frith, 2010; Noy, Dekel, & Alon, 2011) and sometimes showing a benefit of role distribution (Richardson et al., 2015; Vesper, Schmitz, Safra, Sebanz, & Knoblich, 2016; Vesper, Van Der Wel, Knoblich, & Sebanz, 2011). The benefits of mutual alignment and role distribution likely depend on task features such as emergent vs. enforced role distribution and congruent vs. incongruent demands for coordination across individuals (Curioni et al., 2019), as well as expertise of co-actors (Noy et al., 2011). Further research is needed to clarify the conditions under which mutual alignment facilitates coordination more than the leader-follower configuration and vice versa, and to what extent these conditions are present within and beyond iterated pure coordination scenarios.

We hypothesized that brief meditation interventions would improve coordination performance, and that such improvements would be associated with changes in subjective thought. Specifically, we predicted that LKM would increase the rate of other-focused thought (as opposed to self-focused) and that FAM would increase on-task thought (as opposed to off-task). It is likely that our failure to find intervention effects is caused by the brief nature of these interventions.

Future research could build on our results to examine not only what factors facilitate coordination performance, but also how task demands change over time. For example, in our task, participants may require more on-task thought in earlier trials as they learn to establish focal points, but in later trials on-task thought may no longer track coordination performance once participants can rely on resource-efficient decision rules instead of cognitively-demanding perspective-taking processes. Thus, identifying changes in task demands over the course of interaction could inform which methods optimally track mechanisms for coordination.

Overall, our findings suggest that subjective thoughts of participants as they engage in tacit coordination processes matter for the outcomes of this coordination. When both participants engage with each other, then coordination is much better than when either participant is more focused on themselves. Future research should find more effective ways to manipulate thoughts than the interventions we used.

## Data Availability

All data and analysis materials are publicly accessible from the following online repository: https://unishare.nl/index.php/s/mKBRMRetStazctA.
